# Exploring molecular and modular insights into space ionizing radiation effects through heterogeneous gene regulatory networks

**DOI:** 10.1038/s41526-025-00508-6

**Published:** 2025-07-18

**Authors:** Mengqin Yuan, Tao Zhang, Haizhou Liu, Min Long, Quan Wang, Wei Jiang

**Affiliations:** 1 Fujian Provincial Key Laboratory of Precision Medicine for Cancer, the First Affiliated Hospital, Fujian Medical University, Fuzhou, China; 2 National Regional Medical Center, Binhai Campus of the First Affiliated Hospital, Fujian Medical University, Fuzhou, China; 3 Department of Bioinformatics, Fujian Key Laboratory of Medical Bioinformatics, Institute of Precision Medicine, School of Medical Technology and Engineering, Fujian Medical University, Fuzhou, China; 4 Department of Biomedical Engineering, Nanjing University of Aeronautics and Astronautics, Nanjing, China

**Keywords:** Systems biology, Risk factors

## Abstract

Space ionizing radiation is a major risk factor for astronauts, yet its molecular mechanisms remain poorly understood. This study employs an integrative approach to investigate the effects of space ionizing radiation on molecules, modules, biological functions, associated diseases, and potential therapeutic drugs. Using paired samples from five donors subjected to acute ex vivo 2Gy gamma-ray irradiation, we analyzed miRNA and gene expression profiles in human peripheral blood lymphocytes collected 24 h post-exposure, combined with heterogeneous network analysis, identifying 179 key molecules (23 transcription factors, 10 miRNAs, and 146 genes) and 5 key modules. Functional enrichment analysis revealed associations with processes such as cell cycle regulation, cytidine deamination, cell differentiation, viral carcinogenesis, and apoptosis. Radiation was also significantly linked to neoplasms and digestive system diseases. Furthermore, we predicted 20 potential therapeutic compounds, including small molecules (e.g., Navitoclax) and Traditional Chinese Medicine ingredients (e.g., Genistin, Saikosaponin D), which may alleviate radiation-induced damage such as pulmonary fibrosis and oxidative stress. These findings provide novel insights into the molecular mechanisms of space ionizing radiation and may contribute to developing effective strategies to protect astronaut health during space missions.

## Introduction

Many countries are increasingly focusing on space exploration, which stands for a significant future trend in human science and technology. However, astronauts who venture into space face a multitude of environmental stresses, including microgravity, long periods of isolation and confinement, and exposure to space ionizing radiation, among which ionizing radiation from space poses one of the most substantial risks to human health^1^.

In the space environment, ionizing radiation comprises a complex mixture of highly penetrating charged particles from various sources, including galactic cosmic rays (GCR) from outside our solar system, intermittent solar particle events (SPE), and particles trapped in the magnetic field of Earth (i.e., Radiation Belt)^2^. Ionizing radiation can cause significant harm to human body, primarily targeting nuclear DNA, leading to DNA base damage, DNA single-strand breaks (SSB), and DNA double-strand breaks (DSB)^3^. Furthermore, National Aeronautics and Space Administration (NASA) has identified four critical health risks caused by ionizing radiation for astronauts: carcinogenesis, acute radiation syndrome, degenerative tissue changes, and central nervous system (CNS) performance decline^4^. Beyond these concerns, ionizing radiation can also lead to cardiovascular disease and cause damage to the retina and lens, resulting in eye diseases^5^.

The biological effects of ionizing radiation are influenced by multiple factors, including the type of radiation, dose, genotype, tissue type, physiological state, and time after exposure. For instance, Maes et al. demonstrated that multiple miRNAs exhibited consistent expression trends across different radiation doses (0.1 Gy vs. 2 Gy). The magnitude of these changes varied depending on radiation dose and post-exposure time points (0.5, 2, 6, and 24 h), highlighting dose- and time-dependent patterns in miRNA regulation^6^. Similarly, An et al. reported significant differences in miRNA profiles in IM9 lymphoblastic cells exposed to 1 Gy and 10 Gy of ionizing radiation, indicating that higher doses result in more pronounced and sustained molecular alterations^7^. Radiation responses display marked tissue specificity, with different organs exhibiting distinct molecular and biological reactions to identical radiation exposure. This specificity is driven by tissue-dependent regulatory mechanisms, including epigenetic modifications and functional modulation of key factors such as the p53 C-terminus^8,9^. Nevertheless, deep understanding and research on the molecular mechanisms of ionizing radiation remain limited.

Currently, several kinds of radiation biomarkers have been identified, including genes, transcription factors (TF), and microRNAs (miRNA). For example, Shakyawar et al. have unveiled specific genes, such as GADD45, PCNA, CCNA2, CDKN1A, and MDM2, as ionizing radiation exposure-related biomarkers through multi-omics analysis, which can be used to rapidly and cost-effectively detect the extent of ionizing radiation damage^10^. Furthermore, the TF SP1 has emerged as a valuable biomarker of ionizing radiation through participating in the modulation of radiation-induced oxidative stress response pathways and matrix regulation^11^. Additionally, microRNA hsa-miR-133b has been demonstrated as a novel biomarker for human acute radiation syndrome induced by ionizing radiation^12^. However, these biomarkers have predominantly been identified and studied in isolation, neglecting the complex molecular interactions and regulatory networks that underpin radiation-induced cellular responses. Thus, a systems biology approach is warranted to provide a more comprehensive understanding of radiation-perturbed molecular networks.

In this study, we focus on human peripheral blood lymphocytes (HPBLs) and acute 2 Gy exposure to gamma rays. The rationale for selecting HPBLs is their clinical relevance as a surrogate tissue for systemic radiation exposure, given their accessibility and representation of systemic physiological responses. Although 2 Gy is a higher dose than typically encountered during space travel, it serves as a robust model to investigate acute radiation effects, providing a window into the cellular response to significant radiation exposure, as might occur in accidental or therapeutic contexts. Here, we proposed an integrative approach to uncover radiation-perturbed molecular networks, biological function and potential therapeutic drugs. We identified 179 radiation-affected molecules, termed radiation-related key molecules, which can serve as ionizing radiation biomarkers. We also obtained 5 radiation-related key modules which can be used to explore the molecular mechanisms of ionizing radiation by performing functional enrichment analysis. In addition, we found that ionizing radiation have a significant impact on some diseases, such as neoplasms and digestive system diseases. Finally, we screened 20 small compound drugs and Traditional Chinese Medicine (TCM) ingredients, including Navitoclax, Genistin and Saikosaponin D, as potential candidate drugs for the treatment of damage caused by ionizing radiation. Overall, this study has uncovered molecules disrupted by ionizing radiation, elucidated the molecular mechanism of ionizing radiation, explored its relationship with diseases, and predicted drugs with the potential to alleviate radiation-induced damage. This study facilitates the understanding of impact of ionizing radiation on the human body, and provide a valuable reference for formulating ionizing radiation protection strategies.

## Results

### Analysis workflow of the study

**Fig. 1:**
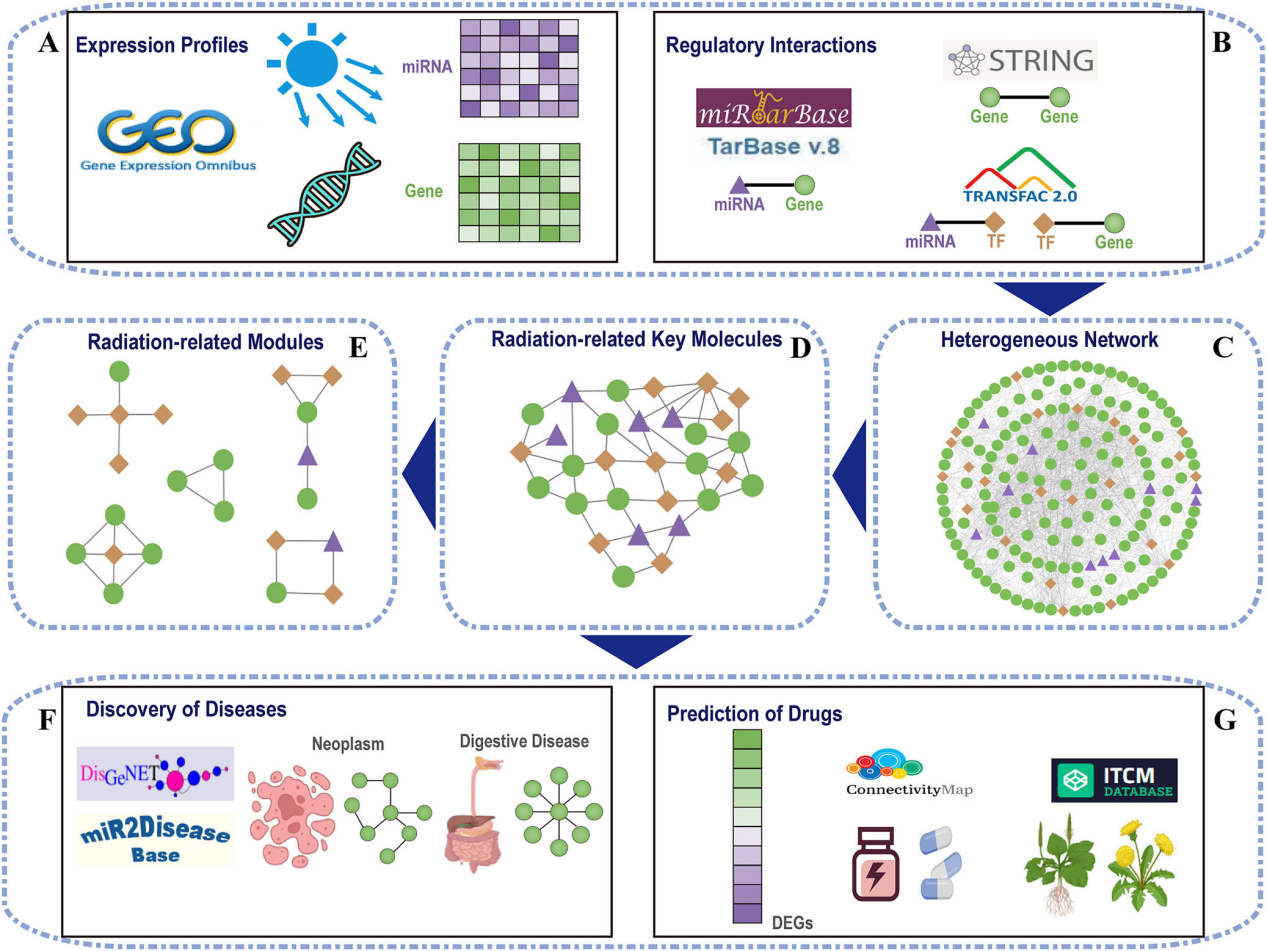
The framework diagram of our study. **A** Obtaining gene and miRNA expression profiles under modeled space ionizing radiation conditions. **B** Collecting the interactions between miRNA and gene, gene and gene, miRNA and TF, and TF and gene. **C** Constructing heterogeneous network through integrating the collected associations between miRNA, TF and gene. **D** Identifying radiation-related key molecules and extracting the subnetwork. **E** Mining radiation-related key modules. **F** Analyzing the relationship between ionizing radiation and diseases. **G** Predicting candidate drugs and TCM ingredients. **C**–**E** were created by Cytoscape (https://cytoscape.org).

The main study process consists of 7 parts (Fig. 1). We first obtained gene and miRNA expression profiles under ionizing radiation conditions from the GEO database (Fig. 1A). We then extracted regulatory relationships between TFs, genes and miRNAs from multiple molecular interaction databases (Fig. 1B) to construct a comprehensive heterogeneous TF-miRNA-gene regulatory network (Fig. 1C). We further identified radiation-related key molecules by integrating differentially expressed molecules (including genes and miRNAs) and TF-miRNA-gene regulatory network using the Random Walk with Restart (RWR) algorithm (Fig. 1D). Based on the subnetwork comprising these key molecules, we applied the Molecular Complex Detection (MCODE) algorithm to identify radiation-related key modules (Fig. 1E). In addition, we found that ionizing radiation is closely related to some diseases, and proposed radiation-disease subnetworks for these diseases (Fig. 1F). Finally, leveraging differentially expressed genes in radiation-related key molecules, we predicted several small compound drugs and TCM ingredients as potential candidates for alleviating ionizing radiation damage (Fig. 1G).

### Radiation-related key molecules and their enriched functions

Our approach took advantage of both the differential expression of biomolecules and the importance of network topology, allowing us to gain valuable insights into the molecular mechanisms underlying the effects of ionizing radiation on biological systems.

**Fig. 2:**
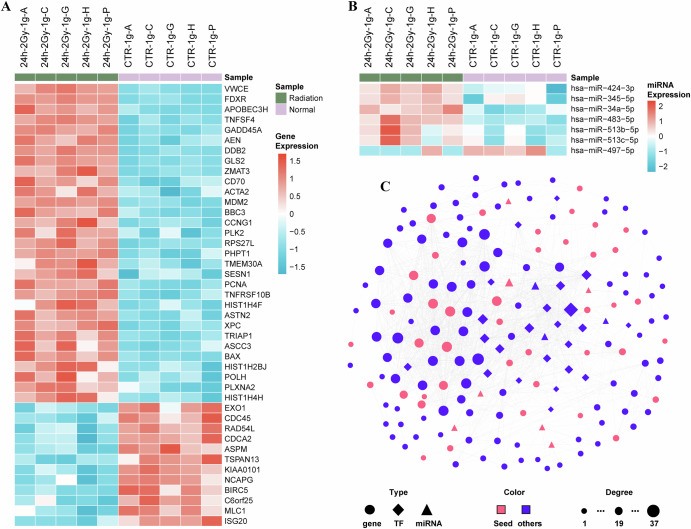
Identification of radiation-related key molecular networks. **A** Heatmap of differentially expressed genes in human lymphocytes after 2 Gy γ-irradiation (|log2FC | >1, adjusted *p* < 0.05; rows: genes, columns: samples). Color represents row-scaled mean expression value (red: upregulation; blue: downregulation). **B** Heatmap of differentially expressed miRNAs (|log2FC | >1, *p* < 0.05; rows: genes, columns: samples). Color represents the same criteria as (**A**). **C** Radiation-related key molecule network derived from comprehensive heterogeneous gene regulatory network incorporating deferentially-expressed genes and miRNAs. The triangle, diamond and circular nodes represent miRNA, TF and gene, respectively. Pink and blue represent seed nodes and non-seed nodes, respectively. The size of the node represents the levels of the node degree.

We performed differential expression analysis on gene and miRNA expression profiles (Supplementary Table 1). We identified 43 significantly differentially expressed genes (adjusted *p* < 0.05 and |log2FC | >1), among which 31 genes were up-regulated, and 12 genes were down-regulated (Fig. 2A). Additionally, seven significantly differentially expressed miRNAs were detected (*p* < 0.05 and |log2FC | >1), among which six miRNAs were up-regulated, and one miRNA was down-regulated (Fig. 2B). To further investigate dose-dependent miRNA responses, we performed a comparative analysis of differentially expressed miRNAs between the 2 Gy and 0.2 Gy (irradiation vs control). Notably, of 7 significantly differentially-expressed miRNAs under 2 Gy exposure, all exhibited concordant expression patterns as 0.2 Gy exposure, suggesting conserved regulatory mechanisms in response to ionizing radiation (Supplementary Fig. 1).

To elucidate potential regulatory mechanisms, we constructed heterogeneous TF-miRNA-gene regulatory network by integrating well-established TF-gene, miRNA-gene, TF-miRNA interactions from several databases, containing 17,939 nodes and 273,659 edges (Supplementary Fig. 2 and Supplementary Table 2). We analyzed topological properties of the constructed network. It exhibits an approximate power-law degree distribution like most biological networks, indicating a scale-free topological property (Supplementary Fig. 3). In the heterogeneous network, we employed the RWR algorithm using these 43 differentially expressed genes and 7 differentially expressed miRNAs as seed nodes. We selected top 1% (179) nodes with the highest RWR scores as the radiation-related key molecules, including 10 miRNAs, 23 TFs and 146 genes (Supplementary Table 3 and Supplementary Data 1). Subsequently, we extracted these 179 radiation-related key molecules and their interactions from the heterogeneous network to form a radiation key molecule network (Fig. 2C).

Several molecules in the radiation key molecule network had been reported to be associated with ionizing radiation. For example, a recent study suggested that hsa-miR-34a-5p could be used as candidate molecules for radiation biomarkers, showing promise on the miRNA regulation of biological effects of ionizing radiation^13^. The transcription factor TP53, with the largest degree in the radiation-related key molecular network, can activate the DNA repair system to maintain the integrity of the whole genome, while the apoptotic process would be initiated if the DNA damage proved irreparable. TP53-governed apoptosis was considered the main cause of ionizing radiation-induced cell death^14^. The gene CHEK1, with the largest betweenness centrality in the network, can restore the mitotic progression in the setting of radiation-induced DNA damage by CHEK1^15^.

**Fig. 3:**
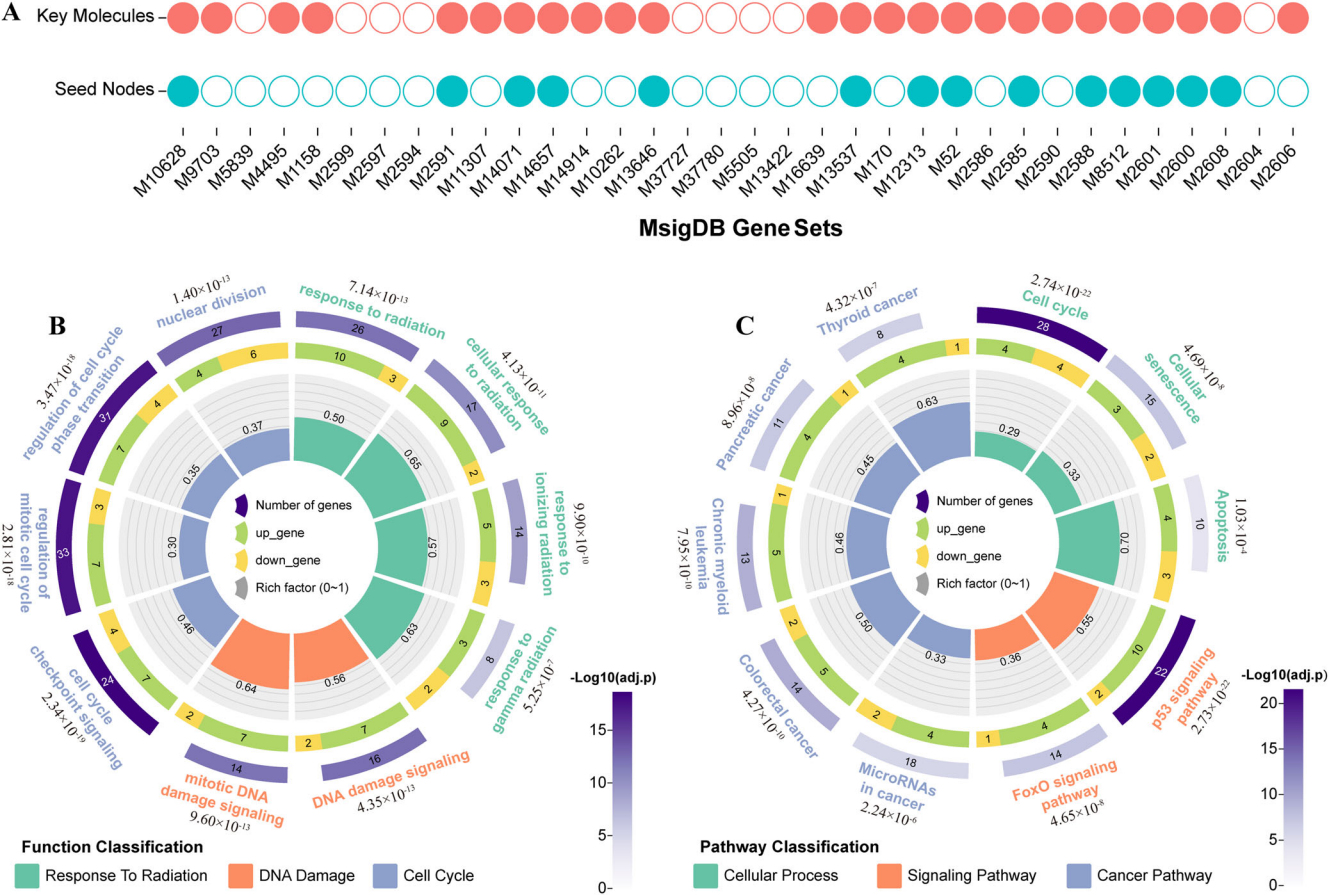
Functional enrichment results of radiation-related key molecules. **A** Bubble plot of enriched terms for the seed nodes (deferentially expressed genes) and radiation-related key molecules. Solid circles indicate significant (hypergeometric test *p* < 0.05), while open circles indicate not significant. **B** Top 10 results of GO functional enrichment map of radiation-related key molecules. **C** Top 10 results of KEGG pathway enrichment map of radiation-related key molecules. The color and number in the outer circle indicate the significance and number of genes enriched in the function or pathway. According to the color key, darker shades represent higher statistical significance. Adjusted *p*-values are provided next to each GO term or pathway. The color and number in the inner circle indicate the number of differentially expressed genes. The height and number of the histogram represent the ratio of differentially expressed genes.

To validate the reliability of the identified radiation-related key molecules, we utilized 34 functional gene sets associated with ionizing radiation from the MsigDB database (https://www.gsea-msigdb.org/gsea/msigdb) as radiation standard gene sets^16^ (Supplementary Table 4). We compared the enrichment results of 43 seed genes and 169 protein coding radiation-related key molecules (comprising 146 genes and 23 TFs) in these radiation standard gene sets. The results showed that the seed genes were significant in 14 standard gene sets, while radiation-related key molecules were significant in 25 standard gene sets (Fig. 3A). The significant improvement of the comparison results indicates that incorporating heterogeneous gene regulatory network is effective, reasonable, and satisfactory.

For the identified radiation-related key molecules, we further explored their biological functions. We performed GO and KEGG enrichment analysis on the genes and TFs among them. GO results indicated that some functions relevant to radiation response are enriched, such as “cellular response to radiation”, “response to ionizing radiation” and “response to gamma radiation”. This may indicate that the identified key molecules play an important role in the effects of ionizing radiation on the body. In addition, we found that these radiation-related key molecules enriched in some biological functions related to DNA damage and cell cycle (Fig. 3B). A previous study has demonstrated that ionizing radiation can trigger DNA damage, thereby inducing cell cycle arrest and various forms of cell death^17^. Furthermore, the results of KEGG revealed that these radiation-related key molecules enriched in some biological pathways related to cell cycle, apoptosis, and cancer (Fig. 3C). Surprisingly, cell cycle appeared in both GO and KEGG results. This suggests that ionizing radiation have important effects on the cell cycle.

Taken together, the radiation-related key molecules identified through our approach held promise as biomarkers for assessing the biological impact of ionizing radiation. Subsequent enrichment analyses could help us deepen knowledge about the mechanisms of ionizing radiation.

### Radiation-related key modules and potential mechanisms

To gain a deeper understanding of the molecular functions and mechanisms of ionizing radiation, we further explored the modules in radiation-related key molecule network.

**Fig. 4:**
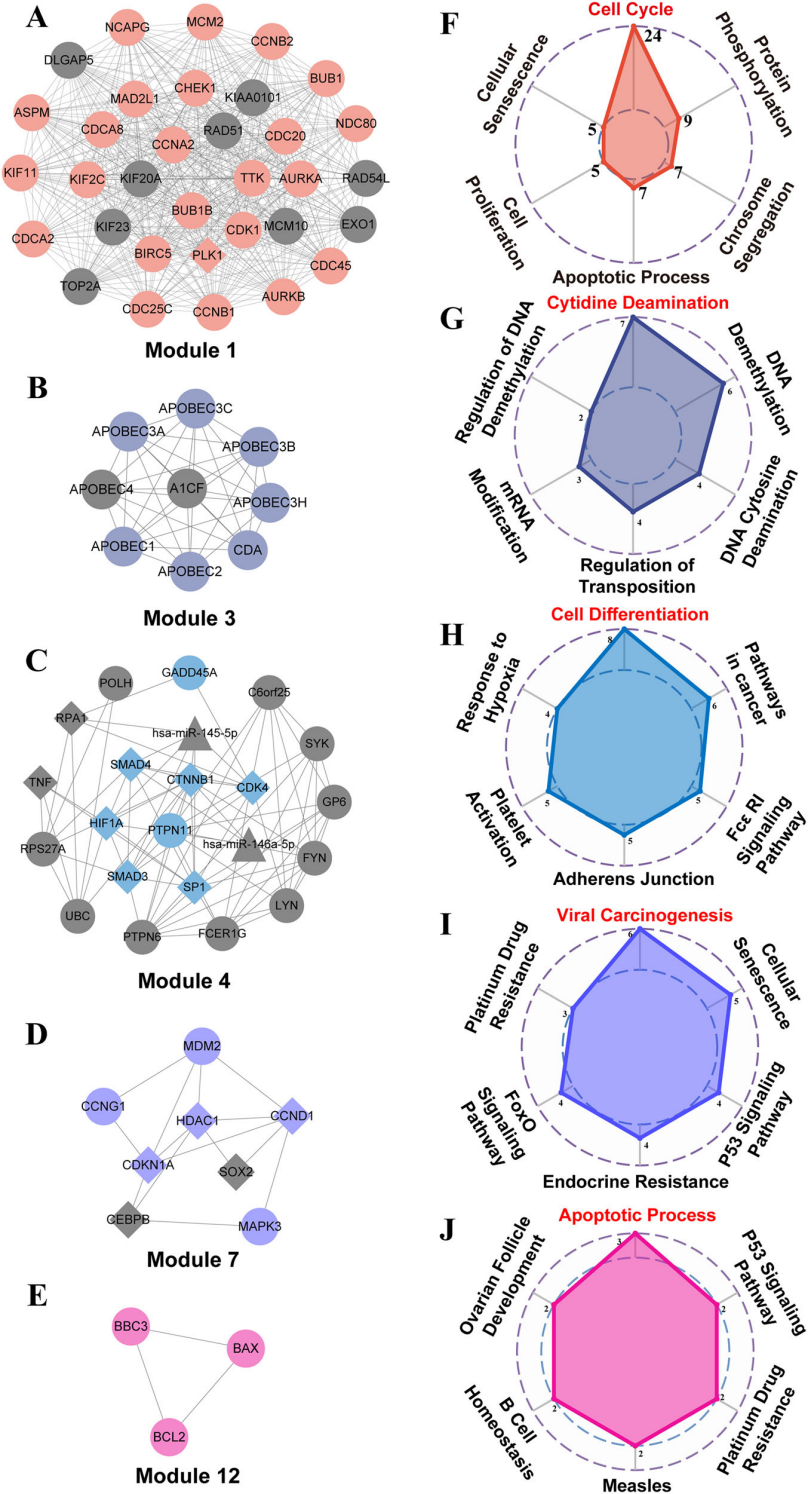
Radiation-related key modules and their functions. **A**–**E** 5 radiation-related key modules, identified by MCODE algorithm and significantly (hypergeometric test, *p* < 0.05) enriched in at least one of the radiation standard gene sets. Highlighted nodes: genes enriched in the module’s top GO term or KEGG pathway. **F**–**J** The top enrichment results of modules in (**A**–**E**), ranked by significance (DAVID, FDR < 0.05). The most significant GO terms or KEGG pathways were marked in red. The number represents the amount of genes enriched in this GO term or KEGG pathway. Only top 6 significant terms shown.

Here, we identified 12 modules by MCODE method (Supplementary Fig. 4). Hypergeometric test was employed to evaluate the enrichment of each module in the radiation standard gene sets. We found five modules exhibited significant enrichment in at least one radiation standard gene set (module 1, module 3, module 4, module 7, and module 12), which were called radiation-related key modules (Fig. 4A–E, Supplementary Fig. 5). We then performed GO and KEGG functional enrichment analysis for the five radiation-related key modules. The six most enriched GO terms or KEGG pathways for each module were shown in Fig. 4F–J.

Module 1 contains 33 genes, among which 24 genes are associated with cell cycle functions. As described above, the cell cycle is closely related to ionizing radiation. Ionizing radiation impacts the cell cycle in two primary ways: by inducing DNA double-strand breaks during interphase, rendering DNA replication impossible without timely repair, and by affecting chromosome formation and movement, disrupting the process of mitosis.

Module 3 stands out with 7 out of 9 genes enriched in functions about cytidine deamination. Cytidine deamination means that cytosine, one of the components of DNA, loses an amino group and becomes uracil. It can lead to base substitution, inversion and DNA chains broken. This may be one of the reasons why ionizing radiation causes genetic mutations and DNA damages.

Within Module 4, 8 out of 22 molecules are enriched for functions related to cell differentiation. Surprisingly, despite many threats of ionizing radiation concerning genomic instability, subjecting cells to the appropriate dosage of ionizing radiation may become a useful method for enhancing directed differentiation in certain stem cell types.

Module 7 reveals that 6 out of 8 molecules are enriched in pathways related to viral carcinogenesis. Viral carcinogenesis occurs when a virus destroys host cell DNA and integrates its genome into the host’s genome, leading to genetic changes and cancer. Ionizing radiation, known for causing DNA damage, may facilitate the integration of oncogenic viruses’ genomes into damaged DNA when the DNA repair system is impaired.

Module 12 comprises three genes (BAX, BCL2, and BBC3) all related to apoptosis. When cells are damaged by ionizing radiation, they can typically repair DNA and restore functionality. However, in cases of severe DNA damage, cells may accumulate irreversible damage, triggering programmed cell death processes like senescence or apoptosis, which serve as self-protection mechanisms.

In summary, our analysis identified radiation-related key modules and elucidated their functions, encompassing cell cycle regulation, cytidine deamination, cell differentiation, viral carcinogenesis, and apoptosis. These findings provide valuable insights for further understanding of the mechanisms of ionizing radiation.

### Radiation-related diseases

**Fig. 5:**
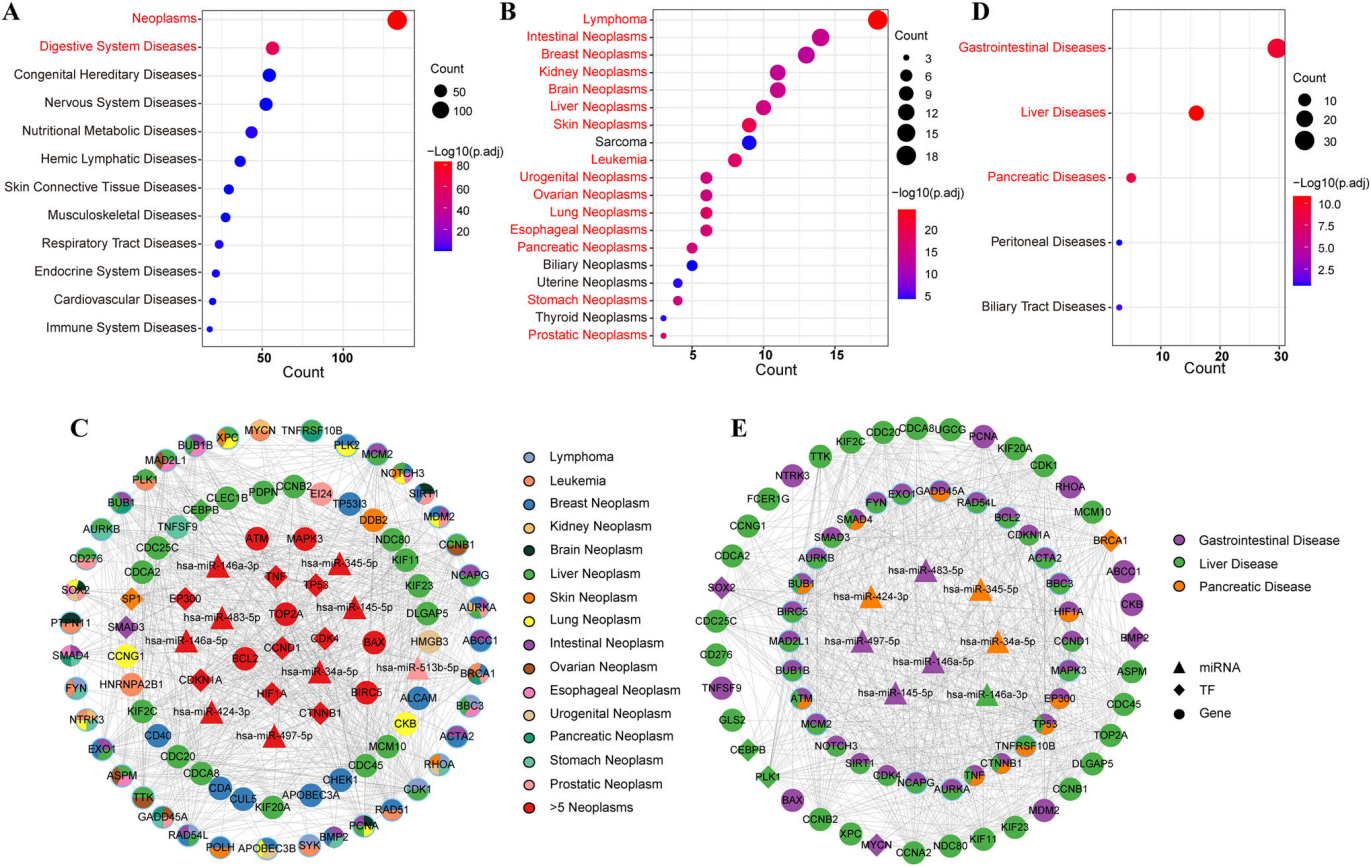
Ionizing radiation-related diseases. **A** Ionizing radiation-related diseases (hypergeometric test, adjusted *p* < 0.05; red: significant). **B** Ionizing radiation-related neoplasm categories. **C** Radiation-neoplasm subnetwork. Red nodes: genes linked to >5 diseases; pie charts: 2–5 diseases; single-color: 1 disease. **D** Ionizing radiation-related digestive system disease categories. **E** Radiation-digestive system disease subnetwork (node scheme as **C**). Disease miRNA and gene data source: miR2Disease and DisGeNET (v7.0).

As stated above, we performed functional enrichment analysis on radiation-related key molecules and modules. We discovered that these molecules and modules enriched in the functions and pathways linked to various diseases and cancers, including viral carcinogenesis and colorectal cancer. Next, we aim to gain a better understanding of the relationships between ionizing radiation and diseases. Our analysis revealed that a total of 439 diseases were significantly enriched in radiation-related key molecules (Details in methods). Based on the MeSH classification system, these diseases fell into 12 distinct categories. A hypergeometric test highlighted the significance of neoplasms and digestive system diseases (Fig. 5A). It is well-documented that ionizing radiation has been associated with tumor development and damage to the digestive system^18^.

Within these diseases, we identified 138 different neoplasm diseases. These diseases were further classified into 19 neoplasm categories based on their anatomical location according to MeSH. Among them, we found that 15 categories of neoplasms exhibited significant relationship with ionizing radiation (Fig. 5B). We extracted neoplasm-related molecules and their interactions from the radiation-related key molecule network, ultimately creating the radiation-neoplasm subnetwork (Fig. 5C). Previous studies have shown that ionizing radiation affects the occurrence and development of neoplasms by disturbing these molecules, such as SP1, BUB1 and hsa-miR-513b-5p^19–25^.

Similarly, radiation-related key molecules exhibited significant enrichment in 57 digestive system diseases. According to MeSH, these diseases were classified into five distinct categories, three of which have significant connection to ionizing radiation (Fig. 5D). We further identified molecules associated with these three categories of diseases from the radiation-related key molecule network, forming the radiation-digestive system disease subnetwork (Fig. 5E). Within this subnetwork, several molecules such as hsa-miR-146a-3p, HIF1A and ATM have been reported to play important roles in ionizing radiation-induced digestive system diseases^26–31^.

In conclusion, our investigation revealed the significant relationship between ionizing radiation and neoplasms and digestive system diseases. Meanwhile, we proposed two radiation-disease subnetworks may facilitate the discovery of underlying molecular mechanisms of ionizing radiation in diseases.

### Candidate drugs for alleviating radiation damage

Ionizing radiation is known to cause varying degrees of harm to human body, potentially leading to a variety of diseases, making it a significant threat to human health. We further predicted the candidate drugs that can potentially alleviate radiation-induced damage based on the identified radiation-related key molecules.

**Fig. 6:**
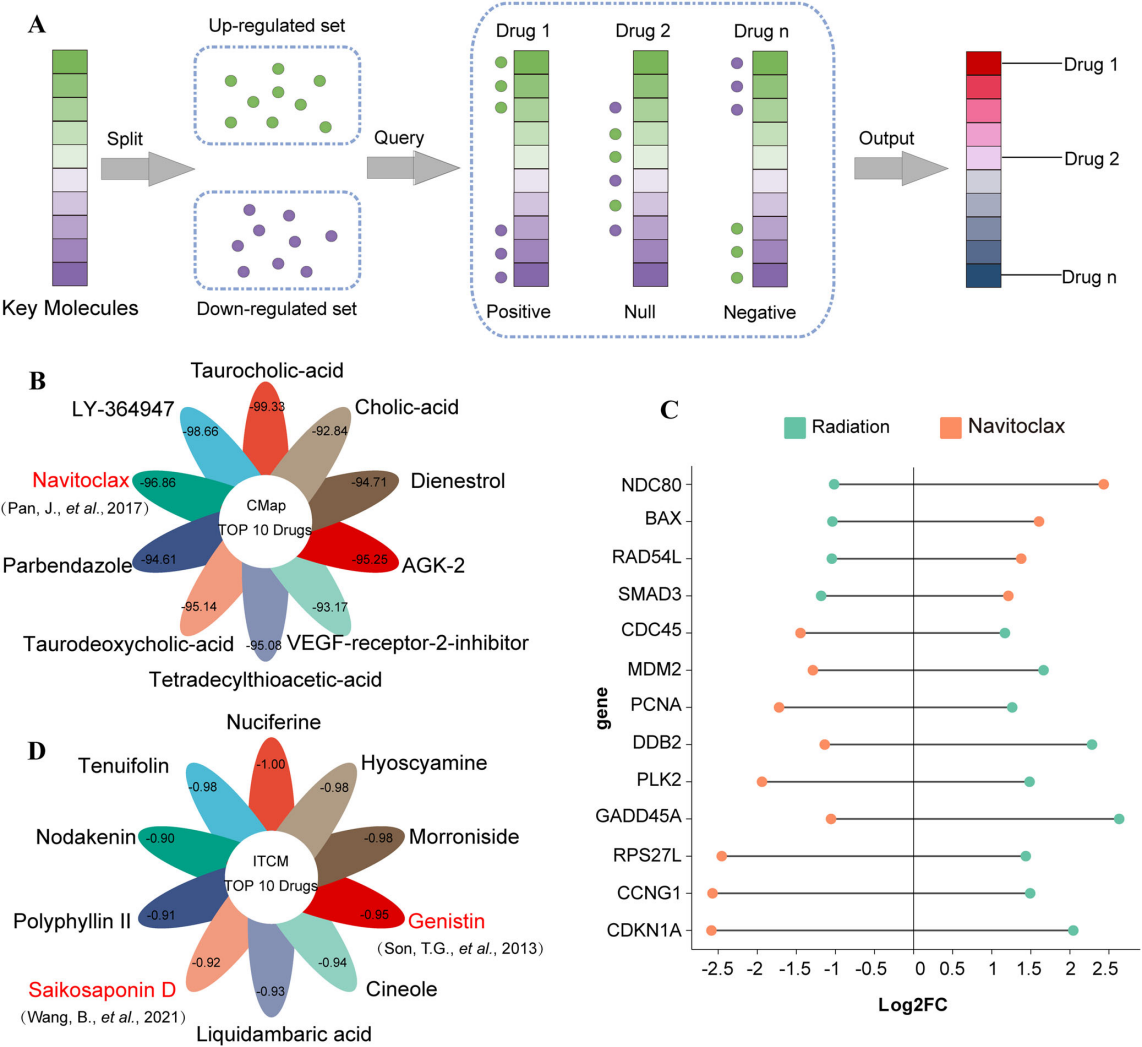
Therapeutic drug prediction for radiation injury based on radiation-related key molecules. **A** Workflow of screening candidate drugs. **B** Top 10 CMap-predicted compounds. Leaf colors represent different drugs: red indicates reported efficacy against radiation damage (referenced in figure), black indicates unreported. Numbers represent CMap connectivity scores (more negative values indicate stronger predicted reversal of radiation-induced gene signatures). **C** Represented radiation-related key genes reversed by the top CMap-predicted compound Navitoclax. Log2FC shows expression changes under radiation/ Navitoclax treatment. **D** Top 10 candidate drugs ITCM-predicted TCM ingredients. Leaf colors follow the same scheme as (**B**). Numbers denote normalized connectivity scores (closer to −1 indicates stronger predicted efficacy).

We used CMap database to predict drugs associated with ionizing radiation (Details in methods). Figure 6A illustrates the core principle of screening candidate drugs using CMap database. It begins with generating molecular signatures through the comparison of radiation samples with normal samples. Subsequently, reverse drug-phenotype relationships are identified by comparing these molecular signatures with drug molecular signatures. When two perturbations produce opposite molecular features, they may have antagonistic effects.

In our study, we employed 76 up-regulated genes and 77 down-regulated genes from the radiation-related key molecules as molecular signatures of radiation phenotypes. According to the result of CMap, we selected the top 10 compounds as the candidate drugs for treatment of radiation injury (Fig. 6B). Previous research has shown that Navitoclax can reverse ionizing radiation-induced pulmonary fibrosis by selectively eliminating senescent cells^32^. As depicted in Fig. 6C, Navitoclax significantly reversed the expression of 13 key radiation-related genes, including BAX, PCNA, CDKN1A, and RAD54L, with log2FC values indicating substantial changes in their expression levels. This reversal of gene expression implies that Navitoclax exerts its protective effects through multiple biological mechanisms: (1) upregulating pro-apoptotic genes such as BAX to remove radiation-damaged cells, (2) downregulating proliferation markers (PCNA) and cell cycle regulators (CDKN1A) to block aberrant cell cycle progression, and (3) modulating DNA damage response genes (RAD54L) to mitigate radiation-induced genomic instability. Collectively, these coordinated molecular changes enable Navitoclax to eliminate senescent cells and inhibit radiation-induced tissue damage^32^.

In addition to the CMap compounds shown above, TCM ingredients have also shown promise in preventing radiation damage. Based on the ITCM platform, we screened 10 candidate TCM ingredients (Fig. 6D). Among these candidates, Genistin exhibits potent antioxidant activity by scavenging oxygen-derived free radicals and suppressing lipid peroxide formation, making it a promising therapeutic agent for preventing and mitigating radiation-induced intestinal injury^33^. Another notable candidate is Saikosaponin D, which has the potential to increase apoptosis by inhibiting mTOR phosphorylation while promoting autophagy^34^.

Through the above analyses, we predicted 10 small compound drugs and 10 TCM ingredients as candidate drugs that can potentially alleviate ionizing radiation damage. Although some of the candidate drugs have not yet been reported to treat the adverse effects of ionizing radiation, they hold promise as novel candidates for protecting against radiation-related harm.

## Discussion

To ensure the health and safety of astronauts during space flights, it is necessary to gain a thorough understanding of molecular mechanisms of ionizing radiation in the space environment and develop effective protective measures. However, the related research is still in its infancy. To address this gap, we proposed an integrative approach to identify radiation-related key molecules through combining differential expression and topological properties in the heterogeneous gene regulatory network. Furthermore, we extracted the radiation-related key modules and explored their biological insights. Finally, the radiation-related diseases and the candidate drugs for alleviating the radiation-induced damage have been predicted and supported by previous studies.

We further compared the biological significance of the radiation-related key molecules with differentially expressed molecules through enrichment analysis in known radiation standard gene set. While the differentially expressed molecules represent immediate radiation-responsive elements, the expanded key-molecule set demonstrated significantly stronger enrichment for radiation-related gene sets and more comprehensive coverage of known radiation response mechanisms. This observation suggests that our network propagation approach successfully captures both the core radiation-responsive elements and their functionally related partners in the molecular network, thereby providing a more complete picture of the radiation response system.

Among the identified hub molecules, hsa-miR-34a-5p, TP53, and CHEK1 play central roles in regulating cellular responses to DNA damage. Notably, our findings regarding miR-34a-5p are consistent with recent experimental work demonstrating its dose-dependent upregulation in peripheral blood mononuclear cell–derived extracellular vesicles and cell type-specific effects on recipient cells, such as senescence in keratinocytes but not fibroblasts^35^. This aligns with the broader observation that while some radiation responses (e.g., ATM-p53 signaling, miRNA modulation) are conserved across different cell types and donors^36–38^, the phenotypic consequences can vary substantially, pointing to tissue- and cell-specific sensitivities^8,9^.

These regulatory elements do not operate in isolation. Network analysis highlights TP53 as a central hub orchestrating multiple stress-response pathways and CHEK1 as a critical bottleneck in post-radiation cell cycle progression. Such network-centric insights complement existing literature, emphasizing that regulatory interconnectivity, not just individual gene expression levels, shapes the outcome of radiation exposure. Importantly, some of the key regulators we identified (e.g., miR-146a-5p) have also been implicated in radiosensitization in cancer therapy, suggesting that molecular responses to radiation may be harnessed to optimize radiotherapeutic strategies.

Furthermore, our results should be interpreted in the context of broader space-specific environmental factors. Microgravity, for instance, can alter immune cell behavior, angiogenesis, and DNA repair fidelity^39,40^. When combined with ionizing radiation, this may produce synergistic or unexpected effects. Similarly, chronic low-dose space radiation—comprising protons, heavy ions, and secondary neutrons—induces complex DNA damage and bystander effects distinct from acute high-dose gamma radiation typically studied on Earth^41–43^.

The relationship between radiation exposure and disease pathogenesis, particularly carcinogenesis, demands rigorous investigation. As a well-established genotoxic agent, radiation induces DNA strand breaks that may culminate in gene mutations or chromosomal aberrations, thereby driving malignant transformation^44^. Notably, Kumar et al. demonstrated that space radiation disrupts pivotal signaling pathways, including Wnt/β-catenin upregulation and concurrent downregulation of Cdc42, Mlck, Par3, and E-cadherin, leading to persistent DNA damage and oxidative stress in intestinal epithelia^45^. Such perturbations, compounded by G2/M checkpoint dysfunction, may foster genomic instability and oncogenic transformation^46^. Crucially, radiation-induced dysregulation extends beyond protein-coding genes. Bisserier et al. identified 27 differentially expressed long non-coding RNAs (lncRNAs) in astronaut-derived exosomes post-spaceflight, suggesting their potential role in aberrant gene regulation and carcinogenic risk^47,48^. These findings underscore the multidimensional impact of radiation, spanning genetic, epigenetic, and post-transcriptional mechanisms.

However, radiation-associated cancer-related gene expression changes do not invariably indicate carcinogenic progression. Many radiation-responsive genes participate in genomic surveillance, with their activation often reflecting protective responses—such as DNA repair, apoptosis, or senescence—rather than malignant transformation. For example, miR-34a-5p, a tumor suppressor in multiple cancers, was shown to promote senescence (not transformation) in irradiated normal keratinocytes, emphasizing context-dependent outcomes^35,39^. This aligns with the paradigm that conserved radiation responses (e.g., miRNA modulation) can yield divergent phenotypic consequences, highlighting tissue-specific vulnerabilities in radiation carcinogenesis.

However, our study still has some shortcomings and limitations in experimental data. First, only five modeled space ionizing radiation samples were analyzed in this study due to the scarcity of experimental data on ionizing radiation in the space environment. To gain a more accurate understanding of the molecular mechanism of ionizing radiation, a larger cohort of samples of space ionizing radiation with high-throughput sequencing data is needed. Second, the expression profiles we downloaded only includes miRNA and gene. If other molecular types of expression data, such as long non-coding RNA (lncRNA) and circular RNA (circRNA), were available in the future, we can explore the mechanisms of ionizing radiation in a more comprehensive way. Third, HPBLs were used to explore the molecular mechanism of ionizing radiation, because the expression data of tissues under space radiation conditions is still not available. Fourth, our current analysis was limited to a single 24-h post-exposure time point and a high-dose (2 Gy) gamma radiation exposure. While this setup provides valuable insights into acute radiation responses, it does not fully replicate the space radiation environment, which typically involves chronic low-dose exposure and a mixed radiation spectrum (e.g., protons, heavy ions, and secondary neutrons). To better characterize the biological effects of space-relevant radiation, future studies should incorporate multiple time points (from acute to chronic exposure), dose gradients (including low, space-relevant doses), and mixed radiation types (e.g., simulated galactic cosmic rays). Finally, while our study identified 20 candidate compounds through CMap-based screening, the novel predictions requiring further experimental verification to confirm their efficacy in mitigating radiation-induced damage. Addressing these aspects will improve our understanding of the temporal dynamics and dose-dependent responses to radiation, ultimately enhancing space radiation risk assessment. We intend to address these aspects in subsequent investigations.

In summary, we revealed the molecular mechanisms of ionizing radiation through a network-based approach; studied the relationship between ionizing radiation and disease, and predicted candidate drugs to alleviate ionizing radiation damage; This study may provide a new reference for understanding the impact of ionizing radiation on the human body, and assist in formulating more effective measures to better protect the health and safety of astronauts during space flights.

## Methods

### Data collection and processing

To investigate the effects of radiation on gene and miRNA expression while controlling for confounding variables, we analyzed paired samples from five human donors under normal gravity (1 g) conditions. Each donor provided both irradiated and non-irradiated (control) samples, resulting in a total of 10 samples (5 irradiated + 5 controls). The gene and miRNA expression data was obtained from Girardi C et al.^40,49^. (Supplementary Table 1). Although the original study included samples exposed to 0.2 Gy radiation for 4 or 24 h, these were excluded from the current analysis due to the lack of corresponding gene expression data. To construct regulatory network between mRNA and miRNA, we focused exclusively on the 2 Gy dose for 24 h group, which provided complete paired molecular profiles (*n* = 10 total samples: 5 irradiated + 5 matched controls). They obtained the human peripheral blood lymphocytes (HPBLs) from five healthy donors, which were incubated in radiation and non-radiation for 24 h, respectively. The ionizing radiation used in the experiment were 2.0 Gy gamma-rays, which were produced by a ^137^Cs source with a dose-rate of 2.8 Gy/min. The expression profiles contain 262 miRNAs and 14,675 genes.

We converted gene probe IDs to gene symbols. If multiple probes corresponded to one gene, we used the average probe expression value as the gene expression value. Finally, the differential expression analysis of miRNAs and genes was completed by the “limma” R package^50^ (https://bioconductor.org/packages/release/bioc/html/limma.html). Differential expression significance was determined via limma with Benjamini-Hochberg (BH) FDR correction to account for multiple comparisons. Genes with adjusted *p* < 0.05 and |log2FC | >1 were considered to be significantly differentially expressed. *P* < 0.05 and |log2FC | >1 were considered to be significantly differentially expressed for miRNAs because no miRNAs were identified when using adjusted *p* < 0.05 as cutoff.

### Construction of the heterogeneous TF-miRNA-gene regulatory network

We constructed a comprehensive heterogeneous TF-miRNA-gene regulatory network composed of TF regulations, miRNA regulations, and protein-protein interactions from several well-established databases. The regulations between TFs and genes were obtained from TRANSFAC Professional database (Release: 2014.2, https://gene-regulation.com)^51^. The regulations between miRNAs and genes were obtained from TarBase v8 (https://dianalab.e-ce.uth.gr/html/diana/web/index.php?r=tarbasev8)^52^, miRTarbase (http://miRTarBase.mbc.nctu.edu.tw)^53^ and TRANSFAC Professional database. In TarBase and miRTarbase, we only retained the regulations that have been validated by low-throughput experiments, such as Western blot and quantitative PCR (qPCR). The regulations between TFs and miRNAs were obtained from TransmiR v2.0 (http://www.cuilab.cn/transmir)^54^ and TRANSFAC Professional database. The protein-protein interactions were obtained from STRING database v12.0 (https://string-db.org)^55^. Only interactions with confidence score greater than 0.7 were selected in our network. Next, we connected all kinds of interactions, and eliminated all self-loops to construct the integrated TF-miRNA-gene heterogeneous network, and visualized with Cytoscape v3.10.0 (https://cytoscape.org)^56^. Collectively, there were 647 TFs, 888 miRNAs and 16,404 genes in the heterogeneous network (Supplementary Table 2).

### Identification of radiation-related key molecules based on RWR algorithm

To systematically identify radiation-related key molecules (including genes and miRNAs), we performed topological analysis of the comprehensive TF-miRNA-gene regulatory network, prioritizing genes and miRNAs that were functionally and structurally proximal to differentially expressed molecules linked to radiation exposure. In this study, the RWR algorithm was used to identify radiation-related key molecules (TFs, miRNAs, and genes) in the heterogeneous network constructed above^57^. By executing the RWR algorithm, the network maintains a balanced state and each node obtains an equilibrium probability, which represents the similarity between the nodes in the network. The formula of RWR is described as Eq. (1):

1 $${{DD}}_{t}={\rm{r}}\times {W}^{m\times m}\times {{DD}}_{t-1}+(1-{\rm{r}})\times {{DD}}_{0}$$

where ${W}^{m\times m}$ is the adjacency matrix of our heterogeneous network. $r$ represents the restart probability. In this study, we set the restart probability $r$ as 0.7. ${{DD}}_{0}$ is the initialization vector with the length of the number of nodes in the network. In ${{DD}}_{0}$, the components corresponding to the significantly differentially expressed molecules (including genes and miRNAs) were set to 1/*n*, where *n* represents the number of seed nodes. The other components were set to 0. ${{DD}}_{t}$ is the vector after ${t}^{{th}}$ updating procedure. The equilibrium probability of each molecule in the network was calculated, then we listed the molecules in descending order based the equilibrium probability. The top one percent of all nodes were finally identified as radiation-related key molecules.

### Detection of modules from radiation-related key molecule network

Radiation-related key molecule network is a subnetwork of the TF-miRNA-gene heterogeneous network. It was obtained by extracting the interactions of all radiation-related key molecules from the heterogeneous network. Modules were identified by the MCODE method, which detects densely connected regions in the radiation-related key molecule network^58^. We used the MCODE plugins (adopted default parameters, https://apps.cytoscape.org/apps/mcode) on the Cytoscape platform and selected the modules with a score larger than 3.

### Functional enrichment analysis

The TFs and genes in radiation-related key molecule were used as input for functional enrichment of Gene Ontology (GO) terms as well as Kyoto Encyclopedia of Genes and Genomes (KEGG) pathways using the ClusterProfiler R package (v4.7.1.003, https://github.com/YuLab-SMU/clusterProfiler)^59^. We performed GO and KEGG enrichment analysis of the TFs and genes in identified modules using The Database for Annotation, Visualization and Integrated Discovery (DAVID, https://davidbioinformatics.nih.gov/)^60^. The GO terms and KEGG pathways with BH FDR < 0.05 were regarded as significant.

### Discovery of radiation-related diseases

The diseases associated with miRNAs in radiation-related key molecules were retrieved from the miR2Disease Database (http://www.mir2disease.org/)^61^. The diseases associated with genes and TFs in radiation-related key molecules were obtained from “Curated gene-disease associations” in DisGeNET (v7.0, http://www.disgenet.org)^62^. The diseases were classified based on MeSH (Medical Subject Headings). Hypergeometric test was used to evaluate the significance of the association between radiation and human diseases. BH adjust *p* < 0.05 was utilized as cutoff to identify significant radiation-related diseases.

### Drug repositioning for alleviating the adverse effects of radiation

We used the Connectivity Map (CMap, https://www.broadinstitute.org/connectivity-map-cmap) to predict drugs by measuring the associations of gene expression patterns between radiated donors’ HPBLs and the reference compound-perturbed cells^63^. The CMap-based approach identified potential therapeutic compounds capable of reversing radiation-associated key gene expression signatures. We selected the top 10 compounds that have significant negative connectivity map scores as candidate drugs. Furthermore, we leveraged an online platform, Integrated Traditional Chinese Medicine (ITCM, http://itcm.biotcm.net/), to search for potential TCM that might be useful against radiation damage^64^. This platform applies the same connectivity mapping principle as CMap but focuses on TCM ingredients. Similarly, we also selected the top 10 TCM ingredients with the most significant negative scores as potential candidates for alleviating ionizing radiation damage.

### Definition of Key Terms

Given the complexity of the multi-network analysis, we provide definitions for critical terms used in this study:

TF-miRNA-gene regulatory network: A heterogeneous network integrating well-established TF-gene, miRNA-gene, and TF-miRNA interactions from databases.

Radiation-related key molecule: High-priority molecules (genes/miRNAs) ranked in the top 1% by RWR scores, identified through network propagation analysis using radiation-responsive differentially expressed molecules as seeds in the TF-miRNA-gene regulatory network.

Radiation-related key network: A subnetwork of radiation-related key molecules and their direct interactions derived from TF-miRNA-gene regulatory network, representing core radiation-perturbed regulatory circuits.

Module: Densely interconnected molecular clusters identified via MCODE analysis within radiation-related key network.

Radiation standard gene set: A curated collection of 34 gene sets related to ionizing radiation (IR) response, systematically retrieved from the MSigDB database using “ionizing radiation” as the search keyword. These gene sets underwent expert-guided screening to ensure biological relevance, serving as a reference for radiation-related functional analysis.

Radiation-related key module: a subset of MCODE-identified modules, which exhibited significant enrichment in at least one radiation standard gene set.

Radiation-neoplasm subnetwork: neoplasm-related molecules (miRNA from miR2Disease and genes from DisGeNET) and their interactions derived from the radiation-related key molecule network.

Radiation-digestive system disease subnetwork: digestive system disease-related molecules (miRNA from miR2Disease and genes from DisGeNET) and their interactions derived from the radiation-related key molecule network.

## Supplementary information

Supplementary Materials

Supplementary Data 1

## Data Availability

All data generated or analysed during this study are included in this published article.

## Code availability

All codes used in this study are available from the corresponding author on reasonable request.
