# Supplementary material for: Exploring molecular and modular insights into space ionizing radiation effects through heterogeneous gene regulatory networks
Source: NPJ Microgravity. 2025 Jul 18;11:44. doi: 10.1038/s41526-025-00508-6 (PMC12274625; doi:10.1038/s41526-025-00508-6)
Supplement: Supplementary file 1 — Supplementary Materials [file 41526_2025_508_MOESM1_ESM.pdf]

Supplementary Materials

Supplementary Figures

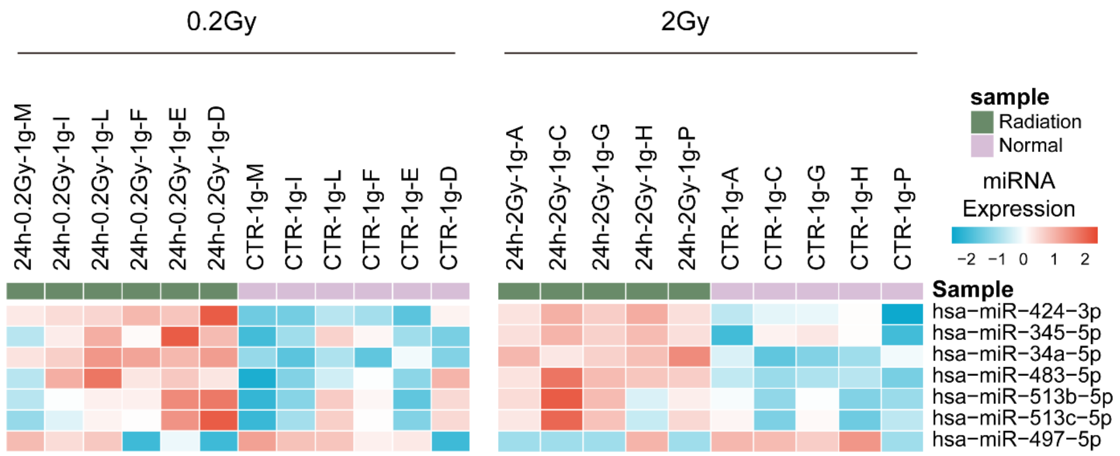

Supplementary Figure 1. The consistent direction of differentially expressed miRNAs (Radiation vs Control) under 0.2Gy and 2Gy. Each sample was named as radiation exposure time-radiation dose-gravity dose-donor number.

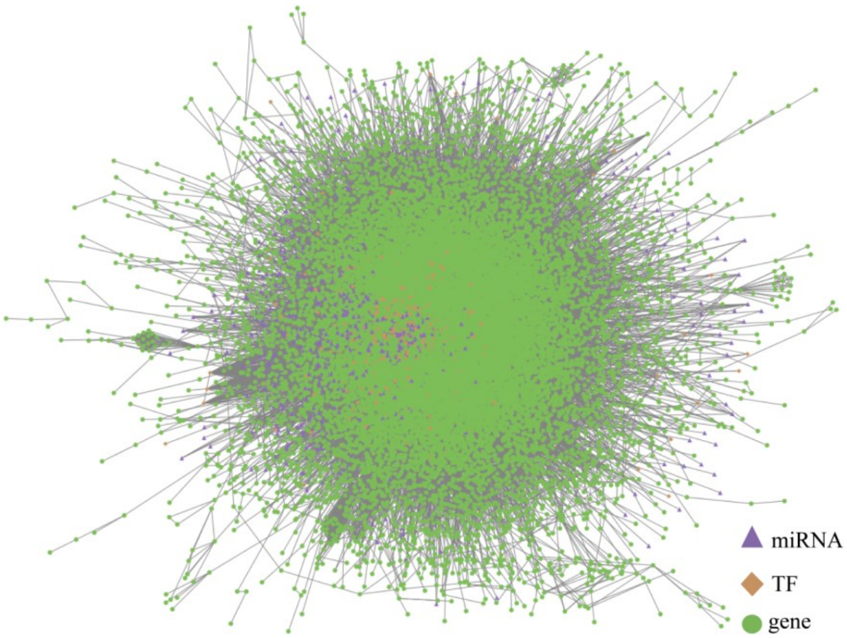

Supplementary Figure 2. The global view of constructed heterogeneous gene regulatory network. The purple triangle, orange diamond and green circle nodes represent miRNA, TF and gene, respectively.

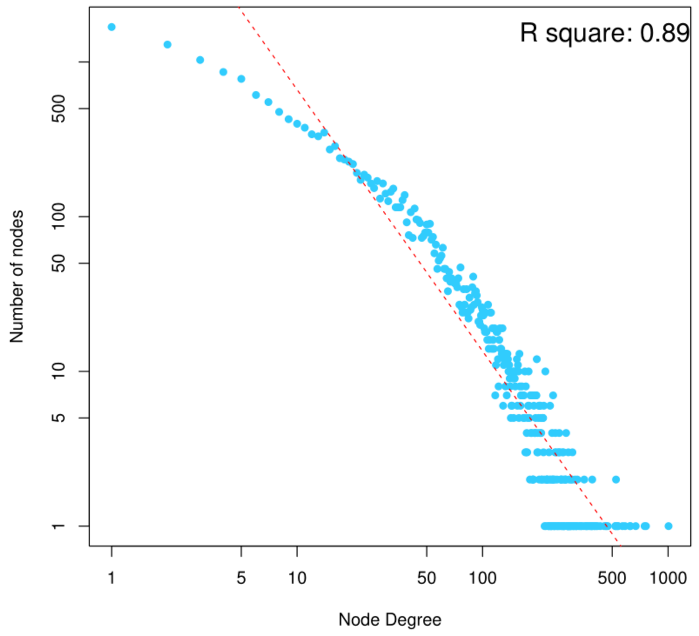

Supplementary Figure 3. The degree distribution of heterogeneous gene regulatory network.

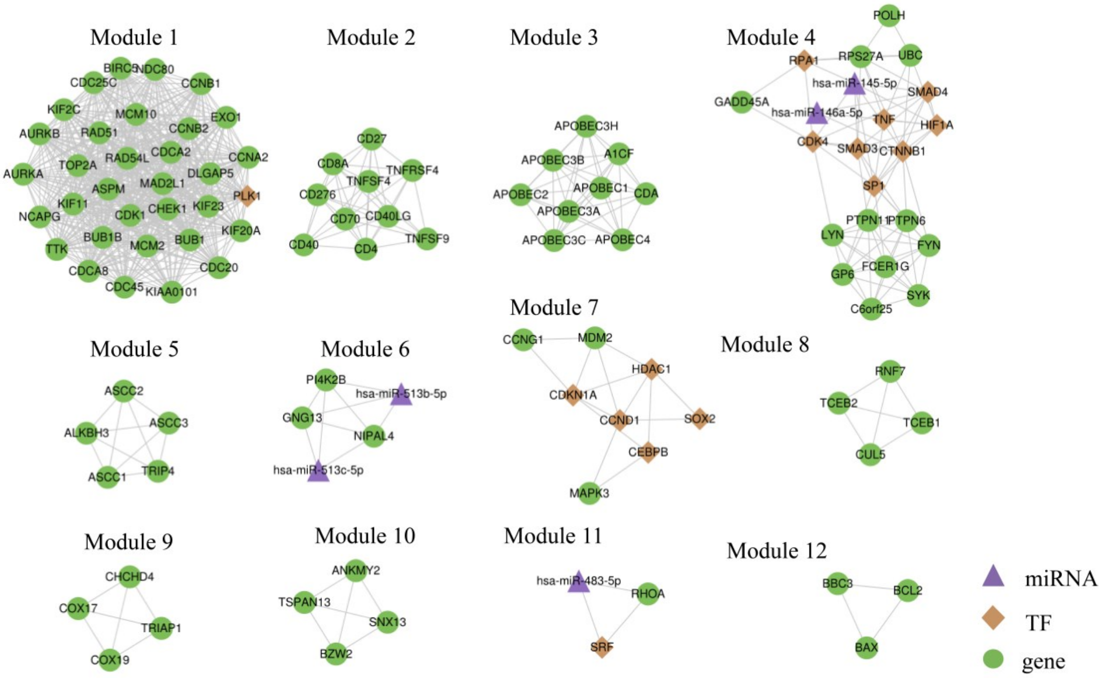

Supplementary Figure 4. 12 modules identified by MCODE approach in radiation-related key molecule network.

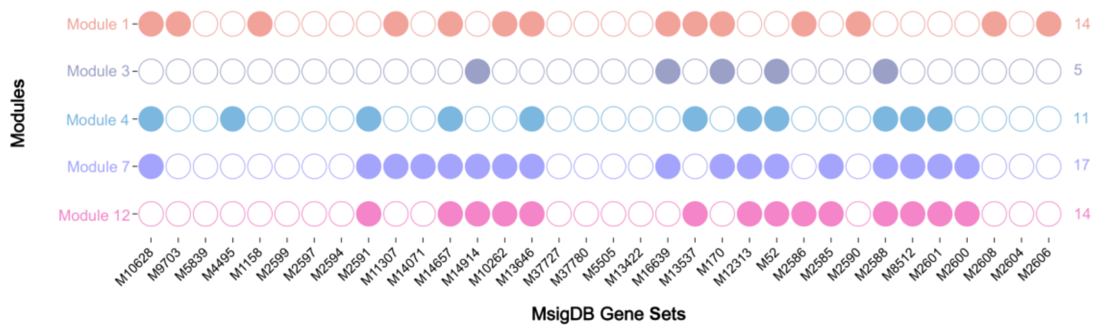

Supplementary Figure 5. Bubble plot for enrichment results of five radiation-related key modules. Solid circles indicate significant (p-value < 0.05) enrichment in radiation standard gene sets, while open circles indicate not significant.

Supplementary Tables

Supplementary Table 1. The samples of miRNA and gene expression profiles.

Data	Radiation miRNA	Radiation gene	Non-radiation miRNA	Non-radiation gene
**Series**	GSE20120	GSE20173	GSE57400	GSE57408
	GSM502123	GSM506087	GSM502349	GSM506089
	GSM502983	GSM506091	GSM502985	GSM506093
**Samples**	GSM502989	GSM506095	GSM502991	GSM506097
	GSM502995	GSM506099	GSM502997	GSM506115
	GSM502091	GSM506102	GSM502114	GSM506116

Supplementary Table 2. Number of interactions in the TF-miRNA-gene heterogeneous network from different resources.

Database	TF-Gene	MiRNA-Gene	TF-MiRNA	Gene-Gene
**TRANSFAC**	4693	2013	79	-
**TarBase**	-	4158	-	-
**miRTarbase**	-	9315	-	-
**TransmiR**	-	-	3921	-
**STRING**	-	-	-	252984
**Total**	4693	12052	3930	252984

Supplementary Table 3. The list of 179 identified radiation-related key molecules.

miRNA (10)	TF (23)	Gene (146)			
hsa-miR-513b-5p	TP53	BBC3	UBC	DLGAP5	GADD45A
hsa-miR-34a-5p	CDKN1A	PCNA	RHOA	TNFSF4	TMEM30A
hsa-miR-513c-5p	MYCN	BIRC5	CKB	PLXNA2	KIAA0101
hsa-miR-483-5p	BMP2	EXO1	UGCG	CD276	TSPAN13
hsa-miR-497-5p	SMAD4	ASPM	TTK	CCNA2	HIST1H4F
hsa-miR-345-5p	CTNNB1	DDB2	ATM	RPS27L	HIST1H2BJ
hsa-miR-424-3p	CCND1	MDM2	A1CF	KIF11	HIST1H4H
hsa-miR-146a-5p	USF1	CDC45	CDA	PTPN6	APOBEC3H
hsa-miR-146a-3p	TNF	CD70	NEMF	CD40LG	C6orf25
hsa-miR-145-5p	CDK4	BAX	CUL5	SLMO1	NIPAL4
	CEBPB	RNF7	MCM2	MCM10	ZNF598
	BRCA1	POLH	BCL2	CDCA8	MRPS25
	SRF	XPC	NCAPG	TOP2A	ANKZF1
	NOTCH3	FDXR	ASTN2	TCEB1	ALKBH3
	SMAD3	GLS2	RAD54L	TCEB2	HMGB3
	HIF1A	AEN	TRIAP1	CDC5L	IL13RA1
	HDAC1	VWCE	ASCC3	PRPF8	KIAA1462
	SP1	PLK2	ZMAT3	KIF23	TSPAN15
	RPA1	MLC1	CDCA2	HSPA4	ANKMY2
	EP300	EI24	ACTA2	NDC80	TNFRSF4
	PLK1	PERP	PHPT1	ASCC2	TMEM30C
	SOX2	CTNS	CCNG1	TRIP4	ALCAM
	SIRT1	DR1	SESN1	EEF1G	FAM160B2
		YBX2	COX19	ASCC1	MAD2L1
		BTG3	KIF2C	RAD51	APOBEC4
		BZW2	AAMP	RPS27A	APOBEC1
		CDK1	ISG20	TP53I3	APOBEC2
		CD4	SNX13	BUB1B	APOBEC3C
		PDPN	COX17	PTPN11	APOBEC3B
		CD8A	CCNB1	AURKA	APOBEC3A
		CD40	CCNB2	CDC25C	AURKB
		CD27	ABCC1	MAPK3	KIF20A
		FYN	NTRK3	TNFSF9	PRELID1
		BUB1	TWSG1	PI4K2B	TNFRSF10B
		SYK	GNG13	FCER1G	HNRNPA2B1
		LYN	CHEK1	CLEC1B	
		GP6	CDC20	CHCHD4	

Supplementary Table 4. 34 MsigDB radiation standard gene sets.

Gene set ID	Gene set name
M10628	BIOCARTA_ATM_PATHWAY
M9703	BIOCARTA_ATRBRCA_PATHWAY
M5839	COLLIS_PRKDC_REGULATORS
M4495	COLLIS_PRKDC_SUBSTRATES
M1158	CROSBY_E2F4_TARGETS
M2599	GHANDHI_BYSTANDER_IRRADIATION_DN
M2597	GHANDHI_BYSTANDER_IRRADIATION_UP
M2594	GHANDHI_DIRECT_IRRADIATION_DN
M2591	GHANDHI_DIRECT_IRRADIATION_UP
M11307	GOBP_CELLULAR_RESPONSE_TO_GAMMA_RADIATION
M14071	GOBP_CELLULAR_RESPONSE_TO_IONIZING_RADIATION
M14657	GOBP_CELLULAR_RESPONSE_TO_RADIATION
M14914	GOBP_RESPONSE_TO_GAMMA_RADIATION
M10262	GOBP_RESPONSE_TO_IONIZING_RADIATION
M13646	GOBP_RESPONSE_TO_RADIATION
M37727	HP_CHROMOSOMAL_BREAKAGE_INDUCED_BY_ IONIZING_RADIATION
M37780	HP_INCREASED_SENSITIVITY_TO_IONIZING_RADIATION
M5505	HUPER_BREAST_BASAL_VS_LUMINAL_DN
M13422	HUPER_BREAST_BASAL_VS_LUMINAL_UP
M16639	KOKKINAKIS_METHIONINE_DEPRIVATION_48HR_DN
M13537	KOKKINAKIS_METHIONINE_DEPRIVATION_48HR_UP
M170	KOKKINAKIS_METHIONINE_DEPRIVATION_96HR_DN
M12313	KOKKINAKIS_METHIONINE_DEPRIVATION_96HR_UP
M52	MACAEVA_PBMC_RESPONSE_TO_IR
M2586	SMIRNOV_RESPONSE_TO_IR_2HR_DN
M2585	SMIRNOV_RESPONSE_TO_IR_2HR_UP
M2590	SMIRNOV_RESPONSE_TO_IR_6HR_DN
M2588	SMIRNOV_RESPONSE_TO_IR_6HR_UP
M8512	TSAI_RESPONSE_TO_IONIZING_RADIATION
M2601	WARTERS_IR_RESPONSE_5GY
M2600	WARTERS_RESPONSE_TO_IR_SKIN
M2608	ZHOU_CELL_CYCLE_GENES_IN_IR_RESPONSE_24HR
M2604	ZHOU_CELL_CYCLE_GENES_IN_IR_RESPONSE_2HR
M2606	ZHOU_CELL_CYCLE_GENES_IN_IR_RESPONSE_6HR

Supplementary Data

Supplementary Data 1: Expression data for the 179 radiation-associated key molecules.
